# Integrate GWAS, eQTL, and mQTL Data to Identify Alzheimer’s Disease-Related Genes

**DOI:** 10.3389/fgene.2019.01021

**Published:** 2019-10-25

**Authors:** Tianyi Zhao, Yang Hu, Tianyi Zang, Yadong Wang

**Affiliations:** ^1^Department of Computer Science and Technology, Harbin Institute of Technology, Harbin, China; ^2^School of Life Science and Technology, Harbin Institute of Technology, Harbin, China

**Keywords:** Alzheimer’s disease, Mendelian randomization, GWAS, eQTL, mQTL

## Abstract

It is estimated that the impact of related genes on the risk of Alzheimer’s disease (AD) is nearly 70%. Identifying candidate causal genes can help treatment and diagnosis. The maturity of sequencing technology and the reduction of cost make genome-wide association study (GWAS) become an important means to find disease-related mutation sites. Because of linkage disequilibrium (LD), neither the gene regulated by SNP nor the specific SNP can be determined. Because GWAS is affected by sample size and interaction, we introduced empirical Bayes (EB) to make a meta-analysis of GWAS to greatly eliminate the bias caused by sample and the interaction of SNP. In addition, most SNPs are in the noncoding region, so it is not clear how they relate to phenotype. In this paper, expression quantitative trait locus (eQTL) studies and methylation quantitative trait locus (mQTL) studies are combined with GWAS to find the genes associated with Alzheimer disease in expression levels by pleiotropy. Summary data-based Mendelian randomization (SMR) is introduced to integrate GWAS and eQTL/mQTL data. Finally, we prioritized 274 significant SNPs, which belong to 20 genes by eQTL analysis and 379 significant SNPs, which belong to seven known genes by mQTL. Among them, 93 SNPs and 2 genes are overlapped. Finally, we did 10 case studies to prove the effectiveness of our method.

## Introduction

It is estimated that the impact of related genes on the risk of AD is nearly 70%. Importantly, neuronal cell death precedes the appearance of cognitive symptoms for 10 years or more, suggesting that targeted treatment needs to be performed before symptoms appear. Therefore, the identification of AD biomarkers such as genes, RNAs (Jiang et al., 2015; Cheng et al., 2018; Cheng et al., 2019), proteins, and metabolites (Cheng et al., 2019) is critical for early detection and early intervention in AD. In addition, identifying candidate genes and loci can also help us understand the pathogenesis of AD and develop drugs.

Recently, Jansen et al. (Jansen et al., 2019) published his AD GWAS study on natural genetics. The sample size is more than eight times that of Lambert et al. (Lambert et al., 2013) in 2013. Due to the increase in the number of samples, they found nine AD risk loci more than in previous studies. Jansen et al. found that most of the AD-related DNA mutations were located in the noncoding part of the genome in regions that affected gene transcription. It means that combining GWAS data with transcriptional expression data will greatly advance AD research (Cheng et al., 2016).

However, GWAS still has certain limitations. The SNP is not necessarily the true pathogenic locus, but only related to the SNP that actually causes the disease due to the LD. GWAS usually analyzes the edge effects of individual loci while ignoring the interaction of multiple genes in complex diseases (Battle et al., 2014). Therefore, GWAS still cannot fully reveal the genetic susceptibility factors of complex diseases (Cheng et al., 2018). It is only an important part of exploring the genetic etiology of complex diseases (Cheng and Hu, 2018). Therefore, using GWAS data for research, we must first start with the expression of SNP, that is, combined with data affecting gene expression, which can weaken the impact of LD on significance. Then, the interaction of multiple genes is considered, that is, the statistical values of each SNP are revised within the whole genome.

It was found that about 80% of the genetic susceptibility loci detected by GWAS were located in the noncoding region of the genome, suggesting that the pathogenic loci may have regulatory functions on gene expression. An important role of large-scale eQTL research is to be able to prioritize SNP loci (Barral et al., 2012) in GWAS susceptible regions and to infer possible biological mechanisms through the influence of DNA polymers on biological characteristics. At present, many studies have used eQTL analysis as a very effective tool to explain the results of GWAS. Hormozdiari et al. (Hormozdiari et al., 2016) present a probabilistic method named eCAVIAR, which can detect target genes by colocalization of GWAS and eQTL signals. Xu et al. purposed a more powerful method based on PrediXcan and TWAS. It can integrate single set or multiple sets of eQTL data with GWAS.

mQTL is mainly based on the analysis of cis-mQTL, that is, using Beta value of methylation level of CpG locus near a gene as dependent variable, screening all SNP variations in the chromosomal region upstream and downstream of the gene as independent variable and regressing each SNP locus S and methylation level M in this region one by one, so as to obtain SNP loci significantly related to the methylation level of a gene. There is no doubt that methylation affects gene expression. This is very similar to eQTL, both of which can cause changes in expression through mutations in a single locus. Therefore, in recent years, more and more studies have been carried out to screen genes related to traits by combining mQTL with GWAS. Hägg et al. (Hägg et al., 2015) integrated GWAS, eQTL, and mQTL to find out genes which are related to obesity. Pharoah et al. (Pharoah et al., 2013) identified three new susceptibility loci for ovarian cancer by GWAS meta-analysis and verified the result by mQTL.

In our previous paper (Hu et al., 2018), we have identified some AD-related genes by GWAS and eQTL using SMR. There are three points to be improved. Firstly, mQTL should be included to verify and improve our result. Secondly, we used several eQTL datasets in that paper, whereas a meta-analysis method should be used to integrate the datasets, which can improve the accuracy of eQTL’s statistical results. Finally, GWAS datasets should also be integrated into one dataset so that can overcome the difference of statistical power caused by sample size.

## Methods

### SMR

Since Zhu et al. proposed “SMR” in 2016, it has become a common way to identify the genes whose expression levels are associated with a complex trait because of pleiotropy. Using GWAS and eQTL data, SMR could screen trait-related genes. After two years, they applied SMR to mQTL data. They found 7,858 DNAm sites which are related to 14 complex traits.

The basic idea of this method is as follows. First, let y be the phenotype, which is the outcome variable. x is the gene expression, which is the exposure factor. z is the gene mutation, which is the instrumental variable. Then, b_xy_ is the effect of x on y, b_zx_ is the effect of z on x, and b_zy_ is the effect of z on y. The definition of b_xy_ is b_xy_ = b_zy_/b_zx_, which means the effect of gene expression on phenotype without confounding factors. This idea is based on the Mendelian randomization (Cheng et al., 2018; Cheng et al., 2019).

**Figure 1:**
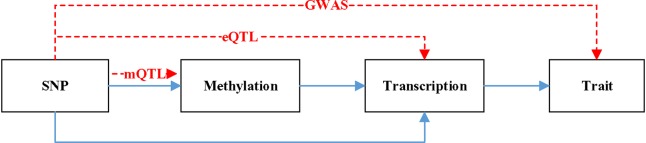
A hypothetical model of a mediation mechanism.

**Figure 1** is a hypothetical model of a mediation mechanism tested in SMR. The blue line represents causal relationship. Methylation will cause SNP. Both SNP and methylation can affect the change of transcription. The change of transcription will cause the difference of trait. The red line denotes the relationship data represents. mQTL denotes the relationship between methylation and SNP. eQTL denotes the relationship between transcription and SNP. GWAS denotes the relationship between SNP and trait.

Based on this hypothesis, many researchers have found the genes which are related to certain traits. Diseases like bone mineral density (BMD) (Meng et al., 2018), amyotrophic lateral sclerosis (ALS) (Du et al., 2017), and neuroticism (Fan et al., 2017) have been found some potential related genes by SMR. Other traits like height, BMI (Yengo et al., 2018), and obesity (Liu et al., 2018) have also researched by SMR.

### Eb-GWAS

Due to the complex linkage effects and statistical errors of the samples, the contribution of GWAS to biological research is reduced. GWAS may associate common diseases with thousands of DNA mutations, that is, every DNA region that happens to be active in diseased tissues may be associated with disease (Jiang et al., 2013). Many GWAS matches are not specifically biologically related to disease and, therefore, cannot be used as effective drug targets. In fact, these “peripheral” mutations are likely to affect the activity of “core” genes, which are more directly related to disease, through complex biochemical regulatory networks (Jiang et al., 2010).

As we discussed before in the introduction, the interaction of multiple genes is considered, that is, the statistical values of each SNP are revised within the whole genome. In this section, we will process GWAS data in two steps: 1. meta-analysis, 2. using EB, revise the statistical value of each SNP within the whole genome.

### Meta-Analysis

Since SE denotes the standard error of each SNP, it represents the reliability of Beta values. Then, weight of each Beta should be:

(1) $$w_{i}=1/{\mathrm{SE}}_{i}^{2}$$

*SE_i_* denotes the standard error for study i, w_i_ denotes the weight of Beta.

Then, the Beta after meta-analysis would be:

(2) $$\beta =\sum_{i}\beta_{i}w_{i}/\sum_{i}w_{i}$$

β_i_ denotes effect size estimate for study i.

Then, we could use the weight of each Beta to calculate the result of meta-analysis.

(3) $$\mathrm{SE}=\sqrt{1/\sum_{i}w_{i}}$$

Finally, the overall Z-score could be obtained by the original equation.

(4) Z=β/SE

### Eb-GWAS

After meta-analysis, we could summary several GWAS datasets into one dataset. Then, we used EB to integrate all the Z scores in the whole genomic level. As we know that the SNP could interact with each other, the Z score of all SNP should have some relationship and obey normal distribution.

The overall Z-score we obtained before obeying normal distribution with standard deviation is 1. Then,

(5) $${\overset{⌢}{Z}}_{i}|Z_{i}\overset{\mathrm{ind}}{∼}N({\overset{⌢}{Z}}_{i},1)$$

${\overset{⌢}{Z}}_{i}$ denotes the Z score we obtained. It is a value with bias. *Z_i_* denotes the real Z score.

Real Z score obeys normal distribution:

(6) $$Z\overset{\mathrm{ind}}{∼}N(\theta ,\sigma^{2})$$

Then, the marginal distribution of ${\overset{⌢}{Z}}_{i}$ is

(7) $$\overset{⌢}{Z}\overset{\mathrm{ind}}{∼}N(\theta ,\sigma^{2}+1)$$

Moreover, the posterior distribution should be:

(8) $$Z_{i}|{\overset{⌢}{Z}}_{i}\overset{\mathrm{ind}}{∼}N(\theta +Β({\overset{⌢}{Ζ}}_{i}-\theta ),B$$

(9) $$B=\frac{\sigma^{2}}{1+\sigma^{2}}$$

Then, we could know that $E({\overset{⌢}{Z}}_{i})=\theta$, so the mean of ${\overset{⌢}{Z}}_{i}$ can be used to estimate θ.

(10) $$\overset{⌢}{\theta }=mean({\overset{⌢}{Z}}_{i})={\bar{\overset{⌢}{Z}}}_{i}$$

(11) $$\frac{\sum_{i}^{N}{\left({\overset{⌢}{Z}}_{i}-{\bar{\overset{⌢}{Z}}}_{i}\right)}^{2}}{\sigma^{2}+1}=\frac{S}{\sigma^{2}+1}∼\chi^{2}(N-1)$$

Then,

(12) $$\frac{\sigma^{2}+1}{S}∼\mathrm{inverse}-\chi^{2}(N-1)$$

From the properties of inverse chi-square distribution,

(13) $$E(\frac{\sigma^{2}+1}{S})∼\frac{1}{N-3}$$

Then,

(14) $$E(\frac{N-3}{S})=\frac{1}{\sigma^{2}+1}=1-B$$

Therefore, the EB estimation of B is

(15) $$B=1-\frac{\left(N-3\right)}{S}$$

Finally, we can put the (Hu et al., 2018) into (Battle et al., 2014)

(16) $$Z_{i}=\bar{\overset{⌢}{Z}}+(1-\frac{\left(N-3\right)}{S})({\overset{⌢}{Z}}_{i}-Z)$$

Then, we have done the meta-analysis and revised the statistical value of each SNP within the whole genome.

### Dataset

As shown in **Table 1** we obtained five GWAS datasets, three eQTL dataset, and three mQTL datasets. All the eQTL and mQTL are from brain tissue. Yang Jian et al. have already meta-analysis the eQTL and mQTL datasets. Therefore, we used the data they processed.

**Table 1:** Datasets used in this paper.

Data	Name	Reference
GWAS	ADNI_DPS_GWAS ADNI_amyloid_GWAS ADNI_hippo_GWAS	Scelsi et al. (2018) (include three datasets)
	IGAP_stage_1	Lambert et al. (2013)
	UK_Biobank	Marioni et al. (2018)
eQTL	GTEx-brain eQTL	GTEx Consortium (2017)
	CMC	Fromer et al. (2016)
	ROSMAP	Ng et al. (2017)
mQTL	ROSMAP	Ng et al. (2017)
	Human fetal brain	Hannon et al. (2016)
	Frontal cortex	Jaffe et al. (2016)

For GWAS dataset, Scelsi M A et al. obtained the data from 1,517 Caucasian ADNI subjects. Lambert JC et al.’s dataset is consisted of 17,008 Alzheimer’s disease cases and 37,154 controls. Marioni R E et al. obtained data from 314,278 participants.

For eQTL dataset, SNPs within 1Mb distance from each probe are available in these three datasets. After meta-analysis, the estimated effective sample size n = 1194.

For mQTL dataset, 5kb, 500kb, and 20kb are the available distance for the three datasets, respectively. After meta-analysis, the estimated effective sample size n = 1160.

## Results

### Results of GWAS Meta-Analysis

We did a meta-analysis of five groups of GWAS data and integrated them into a GWAS file.

**Figure 2:**
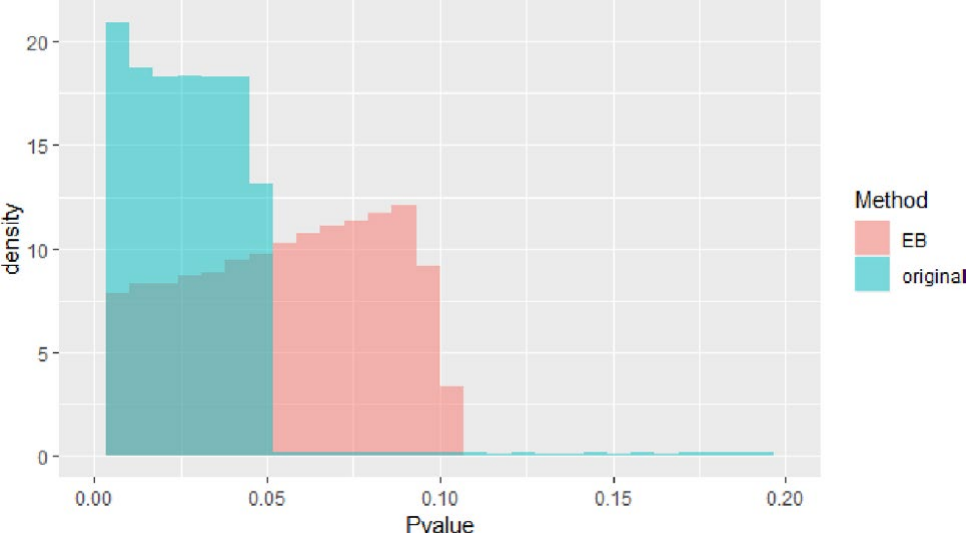
Pvalue density of genome-wide association study (GWAS).

The blue block in **Figure 2** is P value density of GWAS after meta-analysis. The red block in **Figure 2** is P value density of GWAS after EB. As we can see in **Figure 2**, the distribution approximates uniform distribution. After using EB in all SNPs in whole dataset, the P value of the final GWAS data approximates the normal distribution.

### Results of SMR

GWAS included 1,474,846 SNPs, mQTL included 6,966,746, and eQTL included 1,067,443 SNPs. There are 149,326 SNPs occur in both GWAS and eQTL and 408,896 SNPs occur in both GWAS and mQTL. Therefore, we use SMR to test these repeated SNPs in data sets.

Note that some SNPs are marked by multiple probes, so one SNP may significant in more than one gene. One SNP may affect expression of multiple genes.

**Figure 3:**
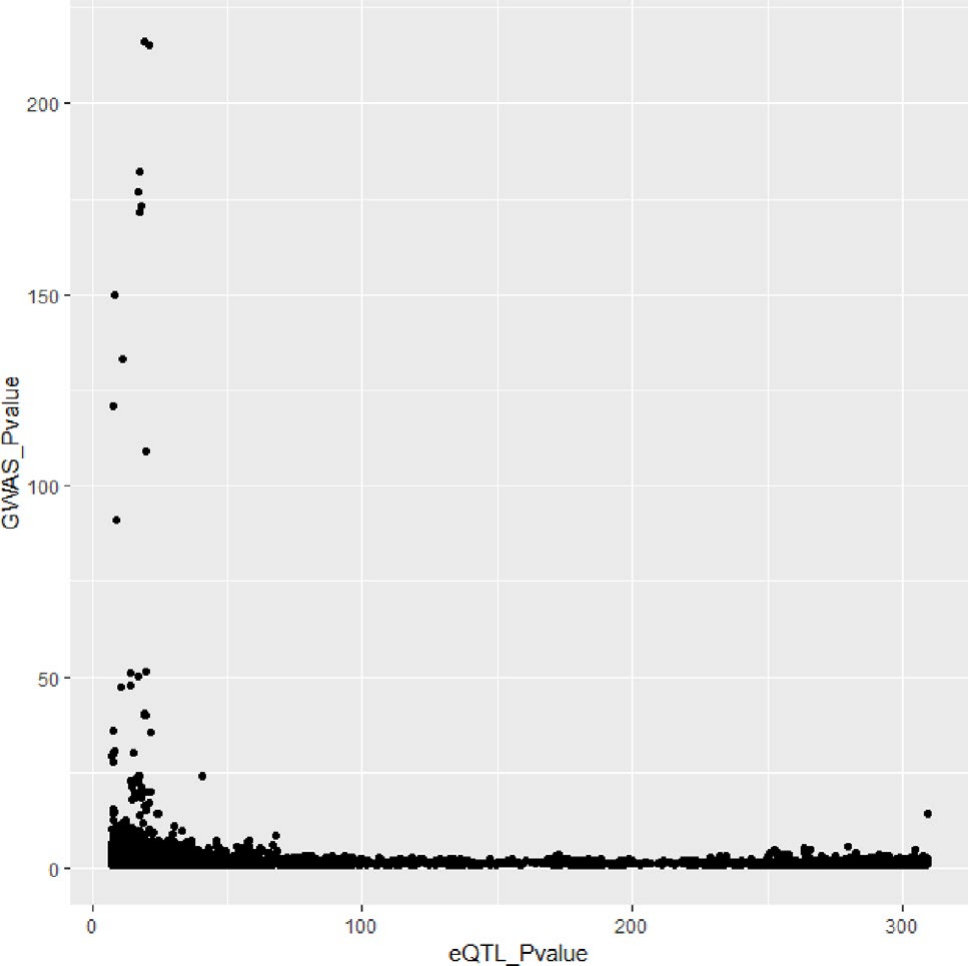
Duplicated SNPs’ P value in genome-wide association study (GWAS) and eQTL.

**Figure 4:**
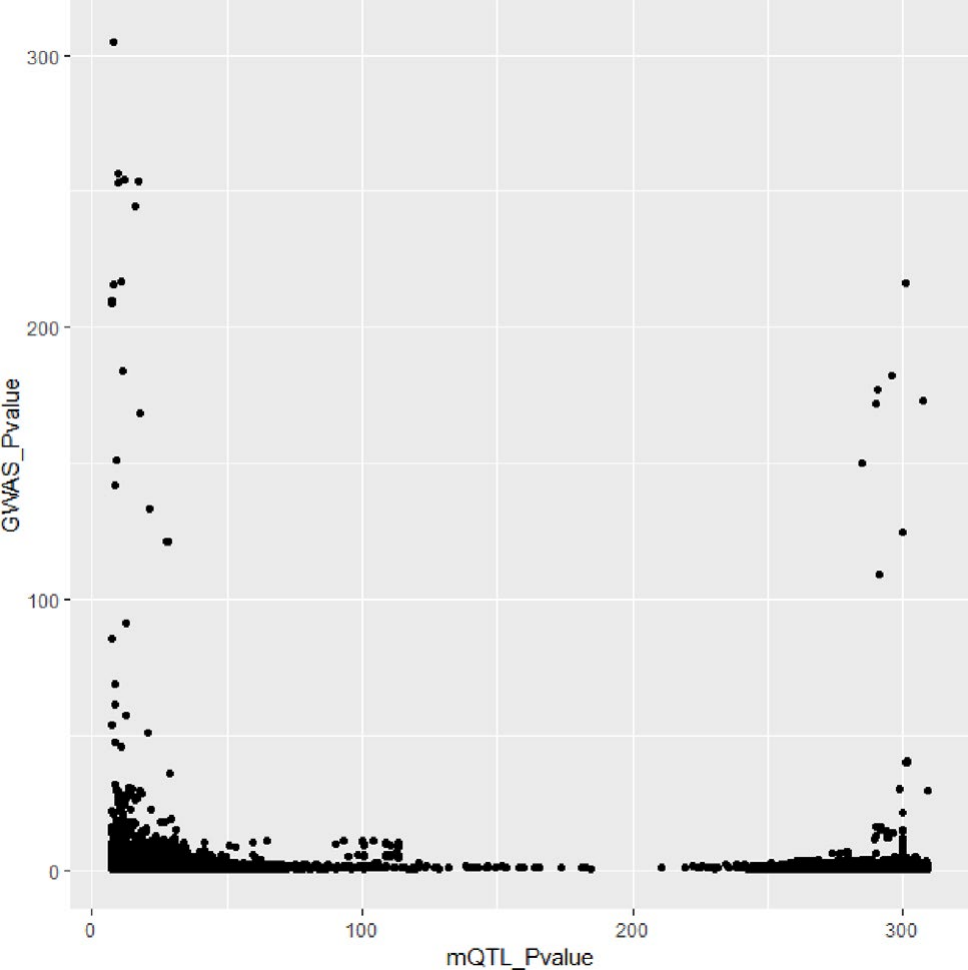
Duplicated SNPs’ P value in genome-wide association study (GWAS) and mQTL.

In **Figures 3** and **4**, we can see that SNPs’ P value in GWAS are not related to eQTL and mQTL. It means that only few significant SNPs in GWAS have significance in eQTL and mQTL. Anyway, the points near the upper right corner in the images mean that the difference in expression level caused by these SNPs is related to AD and SMR can help us detect these SNPs.

We set a threshold as 0.05/(number of probers). For eQTL data, the threshold is 0.05/8362 = 5.98e-06. For mQTL data, the threshold is 0.05/97263 = 5.14e-07. The numbers of SNPs and genes identified by the two experiments are shown in **Table 2**.

**Table 2:** The results of summary data-based Mendelian randomization (SMR).

Dataset	Number of SNPs	Number of Genes
GWAS&eQTL	274	20
GWAS&mQTL	379	7
Overlapped	93	2

**Figure 5:**
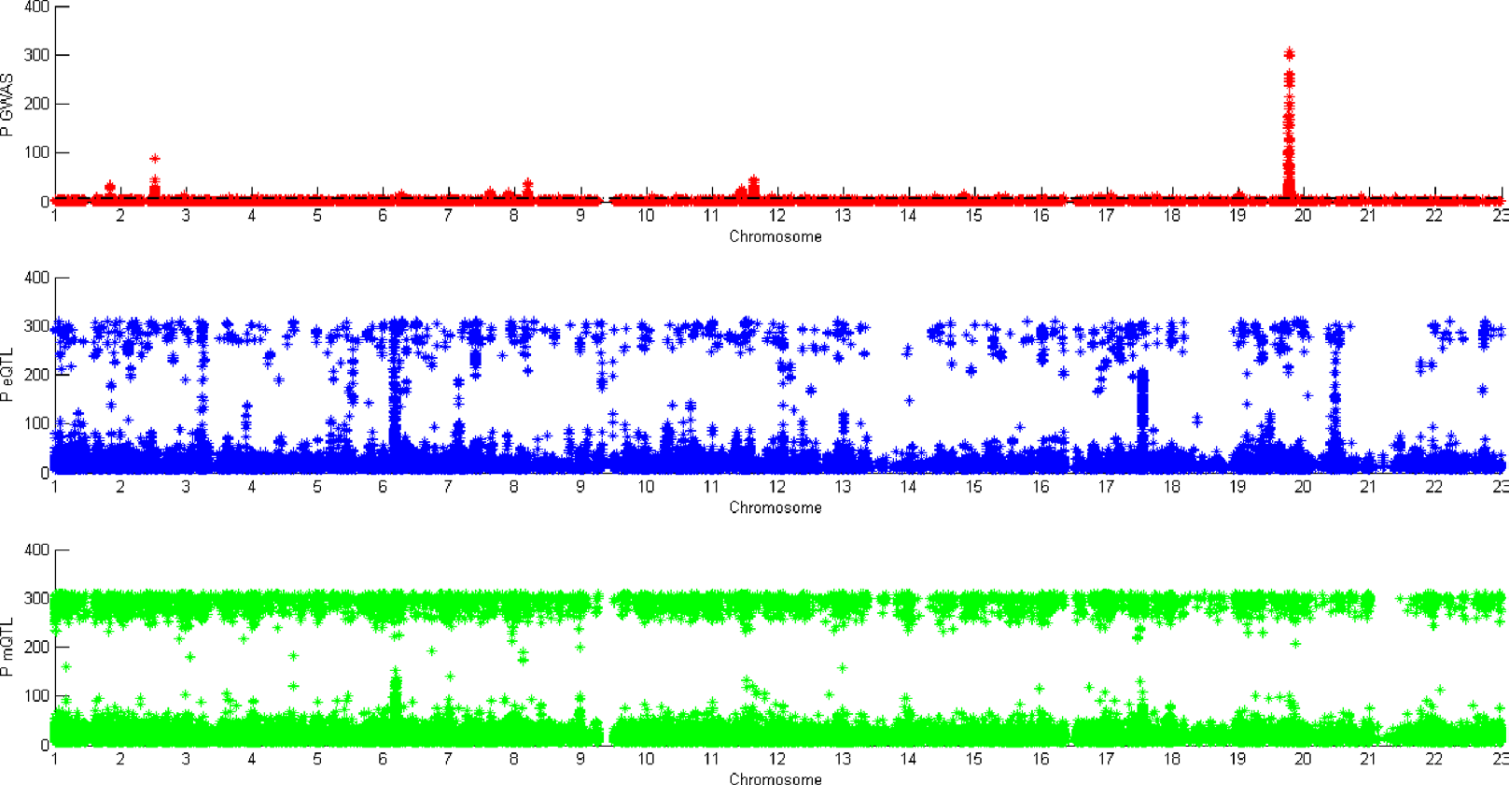
P value of genome-wide association study (GWAS), eQTL, and mQTL.

**Figure 5** shows all the SNPs’ P value. The red points are the P value of GWAS SNPs. The blue points are the P value of eQTL SNPs and the green points are the P value of mQTL SNPs. There is a black line in the first picture. The line is the significant threshold of P value. It is -log10(5*10-8). The SNPs of eQTL and mQTL are already screened so each SNP’s P value is less than 5*10-8.

**Figure 6:**
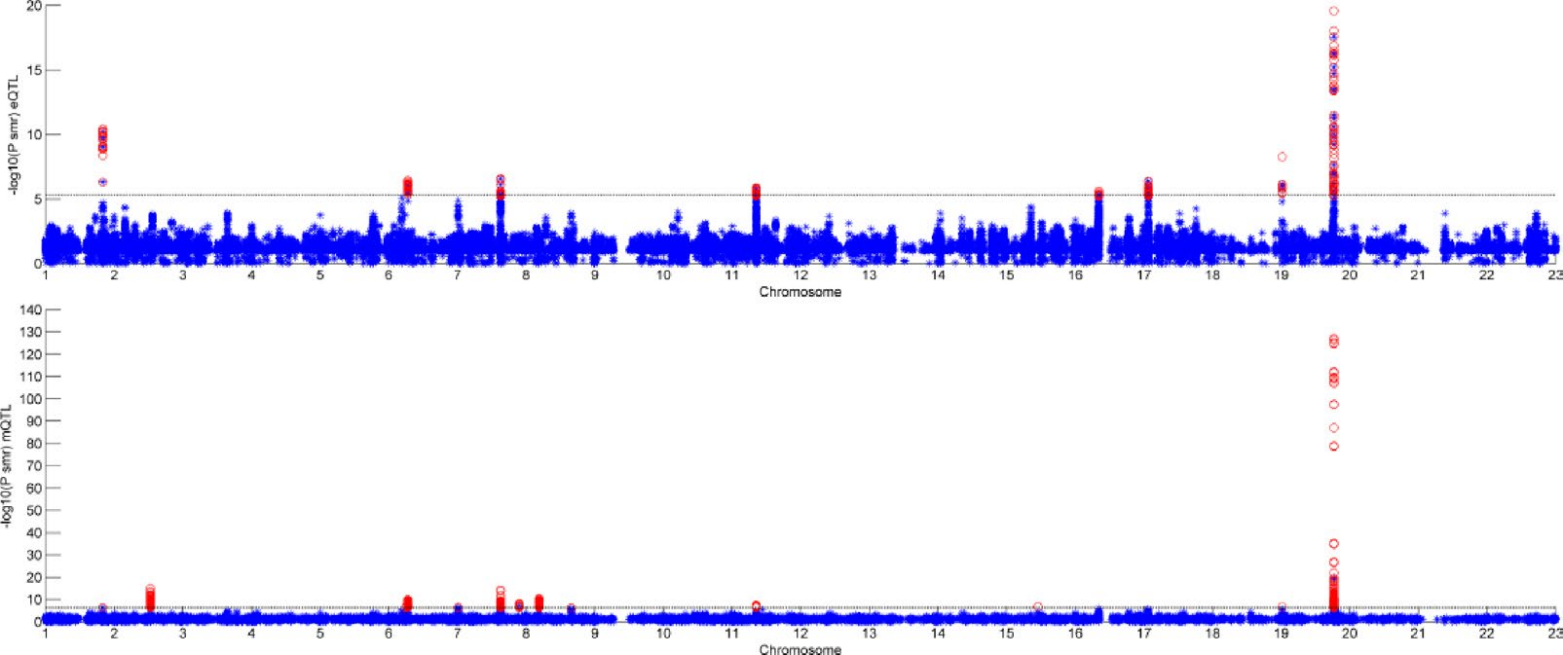
Result of summary data-based Mendelian randomization (SMR).

**Figure 6** shows the result of SMR by two different datasets. The first graph is the result of GWAS and eQTL and the second one is the result of GWAS and mQTL. The black line in the two graphs is significant threshold, respectively. As we can see, only few of SNPs can pass the SMR test. Some of them are not very significant in GWAS, but combined with eQTL or mQTL, they would be significant.

As we can see in **Table 3**, HLA-DQA1 and HLA-DRB5 are selected in both eQTL and mQTL datasets. The HLA complex is located in the 21.31 region (6p21.31) on the short arm of chromosome 6 and is composed of 3.6 million base pairs. It is the region with the highest gene density and the most polymorphic region in human chromosomes. Known as “chemical fingerprints in humans”. Due to the complexity of HLA, the methylation level and expression level differ greatly.

**Table 3:** The candidate genes selected by summary data-based Mendelian randomization (SMR).

	Gene	Number of SNPs
eQTL	*CR1*	20
	*HLA-DRB1*	69
	*HLA-DQA1*	39
	*HLA-DRB5*	8
	*HLA-DQB1*	3
	*HLA-DQB1-AS1*	1
	*RP11-385F7.1*	36
	*ZSCAN21*	8
	*PILRB*	5
	*PILRA*	5
	*MTCH2*	20
	*KAT8*	20
	*AC012146.7*	23
	*ZNF232*	4
	*POLR2E*	7
	*PVR*	12
	*CTB-171A8.1*	24
	*CEACAM19*	11
	*TOMM40*	23
	*ZNF296*	6
mQTL	*BIN1*	11
	*HLA-DRB5*	15
	*HLA-DRB1*	16
	*EPHA1-AS1*	3
	*FAM63B*	2
	*APOC1*	12
	*EXOC3L2*	24

### Case Study

In this section, we want to confirm whether the 25 AD-related genes we found have been reported by others. In order to be precise, we only use the literature that got AD-related genes by biological experiments, rather than the bioinformatics method or GWAS method.

Zhu et al. (2017) found four CR1 SNPs showed significant associations with the Aβ deposition at the baseline level.

James et al. (2018) gathered 71 cognitively healthy women’s the volumes of total gray matter, cerebrocor-tical gray matter, and subcortical gray matter by structural magnetic resonance imaging (sMRI) scan and found that the protective effect of DRB1*13:02 is related to successful elimination of specific pathogens that would ultimately cause gradual brain atrophy.

Yu et al. (2015) found that BIN1 was associated with Aβ load and brain DNA methylation in HLA-DRB5 was associated with pathological AD by 447 participants

Lee et al. (2018) used non-Hispanic Caucasians with neuroimaging and found that HLA-DQB1 is significantly associated with entorhinal cortical thickness by controlling for multiple testing.

Yoshino et al. (2016) found that SNCA mRNA expression in 50 AD subjects was significantly higher than that in control subjects. Therefore, they inferred mRNA expression and methylation of SNCA intron 1 are altered in AD, whereas ZSCAN21 at upstream of these CpG site were reported to bind at intron 1.

Rathore et al. (2018) noted that both TREM2 and PILRB function as activating receptors and signal through DAP12. A reduction of PILRA inhibitory signals in R78 carriers could allow more microglial activation via PILRB/DAP12 signaling and reinforce the cellular mechanisms by which TREM2 is believed to protect from AD incidence.

Ruggiero et al. (2017) did biological experiments on mice and found that MTCH2 is a critical player in neuronal cell biology, controlling mitochondria metabolism, motility, and calcium buffering to regulate hippocampal-dependent cognitive functions.

De Jager et al. (2014) used a collection of 708 prospectively collected autopsied brains to assess the methylation state of the brain’s DNA in relation to AD and found two SNPs associated with POLR2E are related to AD in methylation levels.

Roses et al. (2010) identified polymorphic poly-T variant rs10524523 in transposase of TOMM40 gene, which can be used to estimate the starting age of LOAD with APOE ɛ3 carriers.

Prendecki et al. (2018) recruited 230 individuals and found that APOC1 and TOMM40 rs2075650 polymorphisms may be independent risk factors of developing AD, whose major variants are accompanied by disruption of biothiols metabolism and inefficient removal of DNA oxidation.

We found 10 of 25 genes are reported to be related to AD by biological experiments. Some literary works may found that the other 15 genes are related to AD via other methods, but we would not discuss in this paper. This case study verified the effectiveness of our method and we hope the other 15 genes could be verified by biological experiments in future.

## Conclusion

AD brings great burden to patients and society and identifying AD-related genes can help us known the machanism of AD then diagnose and treatment. In this paper, we used SMR to find AD-related genes by GWAS, eQTL, and mQTL. There are some overlaps between GWAS and the other two datasets, which means that some SNPs are related to AD due to the change of expression level. SMR is a method which can identify the genes whose expression levels are associated with a complex trait because of pleiotropy.

Due to the LD and interaction between genes, GWAS data has bias. In order to overcome these, we did meta-analysis on five GWAS datasets and then used EB to revise the Z-score of each SNPs in whole-SNP level.

Finally, we found 653 SNPs reached the threshold of significance and they are associated with 25 genes. Ninety-three of SNPs are significant in both GWAS&eQTL and GWAS&mQTL tests. We did 10 case studies at last, which means that the 10 of 25 genes we identified have been verified to correlated to AD by biological experiments in existing literary works.

## Data Deposition

### eQTL and mQTL Data

The direct link for accessing eQTL and mQTL data is as follows (origin from PMID: 29891976).

1. eQTL data: https://cnsgenomics.com/data/SMR/Brain-eMeta.tar.gz
2. mQTL data: https://cnsgenomics.com/data/SMR/Brain-mMeta.tar.gz

### GWAS Dataset 1,2,3

GWAS dataset 1,2,3 are from paper PMID:29860282. The direct link is for accessing them is as following.

1. https://www.ebi.ac.uk/gwas/studies/GCST006134 & ftp://ftp.ebi.ac.uk/pub/databases/gwas/summary_statistics/ScelsiMA_29860282_GCST006134
2. https://www.ebi.ac.uk/gwas/studies/GCST006136 & ftp://ftp.ebi.ac.uk/pub/databases/gwas/summary_statistics/ScelsiMA_29860282_GCST006135
3. https://www.ebi.ac.uk/gwas/studies/GCST006135 & ftp://ftp.ebi.ac.uk/pub/databases/gwas/summary_statistics/ScelsiMA_29860282_GCST006136

### GWAS Data 4

GWAS data 4 is from PMID: 24162737. The direct link is for accessing it is as following:

http://web.pasteur-lille.fr/en/recherche/u744/igap/igap_download.php

### GWAS Data 5

GWAS data 5 is from PMID: 29777097. The direct link is for accessing it is as following:

http://datashare.is.ed.ac.uk/download/DS_10283_3364.zip

All code could be downloaded by

https://github.com/zty2009/Integrate-GWAS-eQTL-and-mQTL-data-to-identify-Alzheimer-s-Disease-related-genes

## Author Contributions

TZang and YW are the corresponding authors. They help to revise and support data for this data. TZhao and YH are the co-first authors. They wrote the code and write the paper.

## Funding

This work was supported by the National Natural Science Foundation of China (No: 61571152 and 61502125), the National High-tech R&D Program of China (863 Program) [Nos: 2014AA021505, 2015AA020101, 2015AA020108], the National Science and Technology Major Project [Nos: 2013ZX03005012 and 2016YFC1202302], the Heilongjiang Postdoctoral Fund (Grant No. LBH-Z15179), and the China Postdoctoral Science Foundation (Grant No. 2016M590291).

## Conflict of Interest

The authors declare that the research was conducted in the absence of any commercial or financial relationships that could be construed as a potential conflict of interest.
